# Pericardial Effusion: Overview of Aetiology, Pathophysiology, Diagnosis, and Management

**DOI:** 10.7759/cureus.92177

**Published:** 2025-09-12

**Authors:** Dinesh Abhijeeth Shanker, Akshay Gaur, David Warriner

**Affiliations:** 1 Cardiology and Acute Medicine, Doncaster Royal Infirmary, Doncaster, GBR; 2 Cardiology, Doncaster Royal Infirmary, Doncaster, GBR; 3 Cardiology, Doncaster and Bassetlaw Teaching Hospitals National Health Service (NHS) Foundation Trust, Doncaster, GBR

**Keywords:** cardiac tamponade, emergency pericardiocentesis, pericardial effusion, pericarditis, transthoracic echocardiogram

## Abstract

The pericardium is a membrane that envelopes the heart and the proximal great vessels. In normal physiological conditions, there is approximately 10-50 ml of pericardial fluid. However, accumulation of pericardial fluid can occur due to inflammation or infection of the pericardium and adjacent structures. This may be an asymptomatic and incidental finding following imaging for another disease process, or the patient may present in shock due to cardiac tamponade. It is important to note that it is the rate of accumulation, not absolute size, that primarily determines symptoms. Whilst the clinical suspicion may be raised by the history, examination, electrocardiogram, or chest X-ray, the echocardiogram is the mainstay of diagnosis. Options for treatment will depend on the cause and haemodynamic consequences. This article will consider the aetiology, pathophysiology, investigations and management of pericardial effusions.

## Introduction and background

A pericardial effusion refers to a pathological accumulation of pericardial fluid within the pericardium. The pericardium is a membrane that envelopes the heart and the proximal great vessels. In normal physiological conditions, there are approximately 10-50 millilitres (mL) of pericardial fluid between the layers of pericardium [1]. However, accumulation of fluid can occur due to inflammation, infection, malignancy or systemic diseases. This can impair cardiac function through raised intrapericardial pressures. The clinical presentation may vary; some remain asymptomatic when the discovery of pericardial effusion is an incidental finding, whilst others may present in cardiogenic shock with cardiac tamponade necessitating urgent intervention [2]. Transthoracic echocardiogram (TTE) remains the mainstay of diagnosis, but emerging techniques, such as quantitative fluid analysis and cardiac magnetic resonance imaging (MRI), improve characterisation [3]. Management options hinge on the haemodynamic impact. Stable effusions warrant treatment of underlying causes, whilst tamponade requires urgent pericardiocentesis [3]. Innovations include the pericardial window technique and intrapericardial therapies for malignant effusions [4]. This review synthesises contemporary evidence on the aetiology, pathophysiology, investigations and innovative management of pericardial effusions.

## Review

Anatomy and physiology

**Figure 1:**
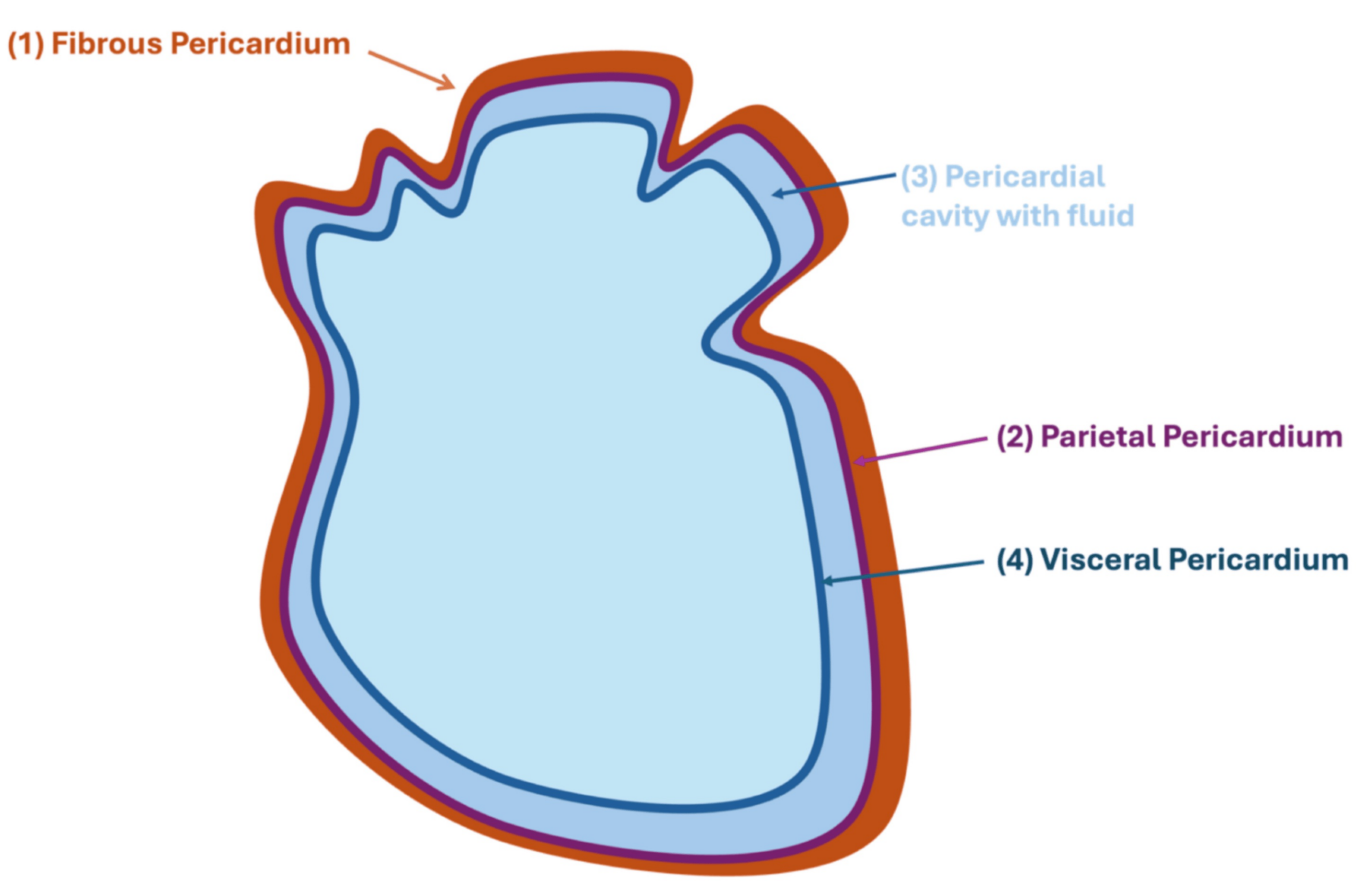
Anatomical illustration of the pericardial layers and cavity. From superficial to deep: (1) fibrous pericardium, (2) parietal pericardium, (3) pericardial cavity, and (4) visceral pericardium This image was created by the authors using Microsoft PowerPoint (Microsoft Corp., Redmond, WA, USA).

Situated in the middle mediastinum of the thorax, the pericardium is a membrane that envelopes the heart and the proximal great vessels. The anatomy and functions of the pericardium and pericardial fluid are summarised in Figure 1 and Table 1 [1,5]. The pericardium is comprised of two layers.

**Table 1:** Functions of the pericardium and pericardial fluid This table was created by the authors.

1)	Limit the distention of the four cardiac chambers
2)	Facilitate ventricular interaction and interdependence
3)	Promote coupling of the atria and ventricles
4)	Equalises physical forces across the myocardium
5)	Minimize friction with surrounding structures
6)	Act as a barrier from the spread of infection

Outer Fibrous Layer

The outer fibrous layer is made up of non-elastic connective tissue, which produces the reflecting image on TTE. It is a crucial layer in protecting the heart from over-expansion, for example, containing an aneurysm. However, due to its inflexibility, a buildup of pericardial fluid leads to increased intrapericardial pressure, the end result of which can be tamponade.

Inner Serous Layer

The inner serous layer is composed of the parietal and visceral layers. The parietal layer lines the inner surface of the fibrous layer, whereas the visceral layer is directly in contact with the heart's surface. The visceral layer, also referred to as the epicardium, is responsible for the production of pericardial fluid. The potential space between these two layers houses the pericardial fluid, which under normal physiological conditions should consist of less than 10-50 mL. This acts as a lubricant, allowing for the frictionless movement of the heart within the mediastinum.

Epidemiology

The likelihood of a pericardial effusion is influenced by the patient’s age, geographical location, and pre-existing comorbidities. Whilst there is little robust data regarding the prevalence and incidence of pericardial effusions, it is estimated that up to 13.6% of admissions into the emergency department with shortness of breath have pericardial effusion with varying degrees of clinical significance [6]. In the Western world, the incidence is estimated to be 3.4%, whilst the prevalence ranges between 5.7% and 9% [7]. In a Mexican study involving 10,653 patients in a tertiary care centre, a prevalence of 3.4% was reported [8].

Aetiology

Any disease process that affects the pericardium can result in pericardial effusion. Numerous causes include infections, trauma, malignancy, and autoimmune and metabolic diseases. It is noted that in the developed countries, up to 50% of the cases are idiopathic [9]. This is followed by infections (15-30%), commonly due to viral pathogens such as adenovirus and coxsackie A and B viruses [10]. Before the antibiotic era, bacterial pericardial effusions frequently arose secondary to systemic infections. The incidence has dramatically decreased with widespread vaccination, effective antimicrobial therapy, and improved cardiothoracic interventions [9].

Infective causes such as tuberculosis (TB) account for nearly 70% of pericardial effusion cases in developing countries [9]. In contrast, the prevalence of TB-related pericardial effusion is only around 4% in developed, non-endemic regions [11]. Approximately 87% of the world’s TB-related pericarditis occurs in Sub-Saharan Africa, the Western Pacific, and South Asia [10]. Among patients co-infected with human immunodeficiency virus (HIV) and TB, up to 90% present with pericardial effusion [11].

Pericardial effusion may be the first sign of disseminated malignancy and is more common in extracardiac cancers such as bone, breast, and/or lymphoma [3]. About 10-25% of non-infectious cases are secondary to cancer [7]. Myocardial infarction (MI)-related pericardial effusion can be present within a few days of an acute MI or as late as six weeks. The classically described Dressler’s syndrome is estimated to have an incidence rate between 3% and 5% [12]. Nearly a third of the patients undergoing cardiac surgery can develop autoimmune-mediated pericardial effusion [3]. In patients with heart failure and cirrhosis, pericardial effusions develop due to raised systemic venous pressure [3]. Autoimmune diseases such as systemic lupus erythematosus, rheumatoid arthritis, and Sjogren’s syndrome are another non-infectious aetiology cause of pericardial effusion, which has an incidence of 5-15% [9].

Since the COVID-19 pandemic in 2020, and less commonly the vaccinations against COVID-19, these have been added to the list of causes of pericardial effusion [10]. Refer to Table 2 for a comprehensive list of causes of pericardial effusion.

**Table 2:** Causes of pericardial effusion HIV: human immunodeficiency virus This table was created by the authors.

Categories	Examples
Idiopathic	Cause unknown
Infections	Bacteria: *Borrelia burgdorferi*, *Chlamydia*, *Haemophilus influenzae*, *Legionella*, *Mycoplasma*, *Mycobacterium tuberculosis*, *Neisseria*, *Pneumococci*, *Staphylococci*, *Streptococci*, *Salmonella*. Viruses: adenovirus, coxsackievirus, cytomegalovirus, Epstein–Barr virus, HIV, influenza virus, Measles-Mumps-Rubella, parvovirus B19. Fungi: *Aspergillus*, *Candida* species, *Histoplasma*. Parasites: *Entamoeba histolytica* (amoebiasis), *Toxoplasma gondii* (toxoplasmosis)
Neoplasm	Primary: angioma, fibroma, lipoma, rhabdomyosarcoma, and teratoma. Secondary: lung or breast carcinoma, lymphoma, and leukaemia
Myocardial infarction	Rupture of a ventricular aneurysm
Drugs	Cyclosporine, heparin, isoniazid, hydralazine, minoxidil, and warfarin
Autoimmune diseases	Inflammatory bowel disease, mixed connective tissue disorder, myasthenia gravis, polyarteritis nodosa, rheumatoid arthritis, systemic lupus erythematosus, scleroderma, temporal arteritis and sarcoidosis
Trauma	Blunt, penetrating or iatrogenic, e.g. perforation caused by catheter insertion, pacemaker implantation or cardiac surgery
Others	Amyloidosis, aortic dissection, hypothyroidism, pericardial injury syndrome, radiation, and uraemia

Pathophysiology

Mechanisms of Accumulation

Pericardial fluid can accumulate due to three main processes: decreased absorption, increased production, or haemorrhage from cardiac injury. In all these conditions, a pericardial effusion can occur if the rate of production is greater than the rate of drainage. There are various terms used when describing pericardial fluid and related disease processes, summarised in Tables 2-3. The accumulation is usually owing to inflammation or infection of the pericardium and/or adjacent structures. Pericardial fluid consists of a plasma ultrafiltrate, and production depends on the interplay of the hydrostatic and osmotic pressures.

**Table 3:** Terminology describing the composition of pericardial fluid This table was created by the authors.

Term	Definition	Description
Serous	Straw-coloured fluid	Pale, yellow, and transparent
Sero-sanginous	Blood-stained fluid	Thin, pink, and watery
Sanginous	Fresh, frank blood	Thick and red
Purulent	Pus	Thick and grey, green, or yellow

Increase in hydrostatic pressure: Conditions that cause an increase in hydrostatic pressure, such as heart failure, or a decrease in osmotic pressure, such as hepatic cirrhosis, lead to an increased volume of pericardial fluid due to reduced resorption, resulting in the accumulation of a transudate. In addition, damage to the lymphatic system can disturb the balance between production and drainage, also leading to the accumulation of fluid.

Increase in production: Conditions that cause inflammation of the pericardium, such as pericarditis, increase vascular permeability and result in the accumulation of an exudate due to increased production of pericardial fluid.

Haemorrhage: Finally, in the case of cardiac trauma, haemopericardium is a typically rapid accumulation of blood in the pericardial space due to perforation of the heart or proximal great vessels.

Impact on Pressure-Volume Curve

In most of these conditions, a pericardial effusion can occur if the rate of production is greater than the rate of drainage. There are various terms used to describe pericardial fluid and related disease processes, summarised in Tables 3-4.

**Table 4:** Terminology describing common pericardial disease processes This table was created by the authors.

Term	Definition	Common cause
Pneumopericardium	Presence of air in the pericardium	Gas producing organisms
Haemopericardium	Presence of blood in the pericardium	Trauma or iatrogenic
Chylopericardium	Presence of lymph in the pericardium	Injury to thoracic duct

Pericardial effusions cause haemodynamic compromise based on the rate of accumulation, not absolute volume. Slow, global fluid buildup allows pericardial adaptation, whilst rapid and/or localised accumulation overwhelms compensatory mechanisms, precipitating tamponade.

**Figure 2:**
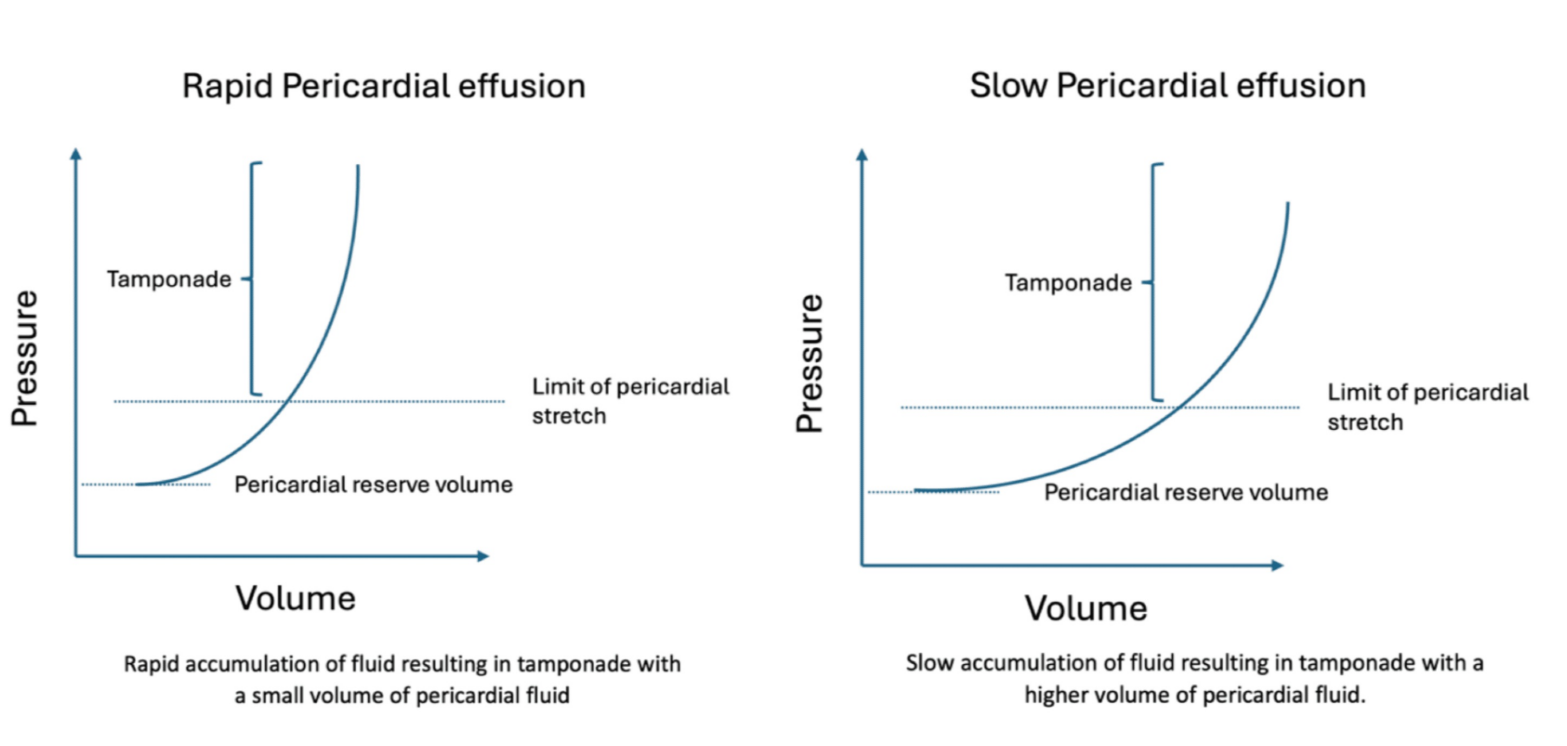
Pressure-volume curves illustrating rapid fluid accumulation (steep curve) cause tamponade with small volumes, whilst slow accumulation (gradual curve) allows adaptation until larger volumes decompensate This image was created by the authors using Microsoft PowerPoint (Microsoft Corp., Redmond, WA, USA). [2]

The pressure-volume curve (Figure 2) is typically flat under normal circumstances; however, when the fluid builds up to the upper limit of normal pericardial reserve volume, the curve shoots up and takes a J shape due to a reduction in the compliance of the sac. For every slight increase in the fluid, there is a dramatic rise in the pressure exerted. Therefore, if the fluid accumulates rapidly, such as in a traumatic tamponade, the implication is that even a slight increase in the fluid could exert significant pressure on the heart, resulting in tamponade.

In acute pericardial effusion, the first chamber to be affected is the right atrium, due to its lower pressures compared to the other chambers. This reduces the cardiac filling during diastole, which is the critical point. Cardiac filling is determined by the transmural pressure, which is the difference between intracardiac pressure and pericardial pressure. A progressive increase in fluid results in compression of the atria and ventricles, which in turn decreases stroke volume and, consequently, cardiac output. This results in adrenergic stimulation, which causes a rise in heart rate through the β-adrenergic receptors to enhance cardiac output, a product of stroke volume and heart rate. Adrenergic stimulation also has an inotropic effect, which improves the ejection fraction. Adrenergic stimulation through alpha receptors causes an increase in systemic and pulmonary venous pressures, which in turn increases the blood volume. These compensatory mechanisms attempt to support the heart until a definitive treatment is sought. However, in the absence of this, the heart may be severely compromised and go into florid tamponade. Patients who are not able to mount an adrenergic response, in instances where they are β-blocked, may be more susceptible to tamponade.

Clinical features

History

Pericardial effusion in isolation and irrespective of size doesn’t always cause symptoms. However, clues to look out for in history, mainly if the effusion is found incidentally, include pleuritic chest pain, shortness of breath, especially on exertion, and a sensation of fullness in the chest. Symptoms secondary to compression of local structures, i.e. dysphagia secondary to compression against the oesophagus or hoarseness in voice due to compression of the recurrent laryngeal nerve, are rare [13]. Non-specific symptoms such as cough, fever (secondary to infection or autoimmune mediation), palpitation, anorexia, and fatigue may also be present, making diagnosis challenging. Once a pericardial effusion is identified, the next step is to ensure the patient is haemodynamically stable and then proceed to identify the likely aetiology.

Examination

On cardiovascular examination, features of pericardial effusion may be unremarkable unless the effusion is large, which may result in muffled heart sounds on auscultation and difficulty palpating the apical pulse. If associated with pericarditis, a pericardial rub may be present. In cardiac tamponade, patients may present with cardiogenic shock, e.g. hypotension and tachycardia, or in cardiac arrest with pulseless electrical activity [14]. It is worth remembering that rate-limited medication may mask any frank tachycardia. Patients in cardiogenic shock will also display signs such as tachypnoea, altered sensorium, peripheral cyanosis, and diaphoresis. In short, cardiac tamponade is a clinical diagnosis, and these patients look very sick. Jugular venous pressure may be markedly elevated with the absence of y descent.

Roy et al. reported pooled sensitivities of 77% for tachycardia, 26% for hypotension, 76% for elevated jugular venous pressure, and 28% for diminished heart sounds [15]. Two classic hallmarks of cardiac tamponade include pulsus paradoxus and Beck’s triad. Pulsus paradoxus refers to an inspiratory drop in systolic blood pressure of 10 mmHg. The sensitivity and specificity of pulsus paradoxus are 98% and 70%, respectively, according to Curtis et al. [16]. Beck’s triad comprises hypotension, muffled heart sounds, and a raised jugular venous pulse. Whilst this may indicate severe tamponade, it is only seen in less than 50% of the patients; therefore, its absence should not rule out tamponade [17].

Investigations

Investigations need to be tailored to confirm the presence of pericardial effusion and identify its aetiology and haemodynamic impact.

Blood Tests

Blood tests are part of the diagnostic workup to determine the underlying cause of the pericardial effusion. As part of initial screening, an elevated white cell count may suggest signs of inflammation or infection. Anaemia can also represent chronic disease processes such as malignancy. Autoimmune screening, including but not limited to anti-nuclear antibody, rheumatoid factor, or anti-double-stranded DNA antibody, also plays a pivotal role in ruling out autoimmune conditions such as rheumatoid arthritis or systemic lupus erythematosus as causes. Examples of blood tests that are conducted as part of diagnosis are complete blood count, renal function, liver function, clotting, procalcitonin, troponin, C-reactive protein, and autoimmune/vasculitis screen.

Electrocardiography

**Figure 3:**
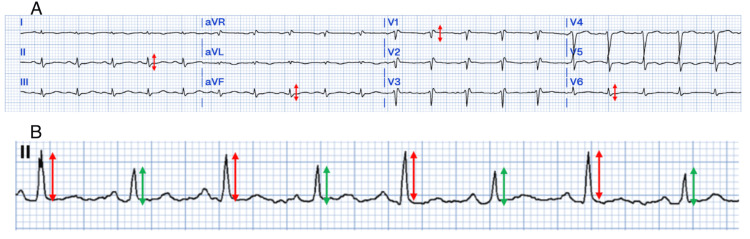
(A) 12-lead electrocardiogram demonstrating low-voltage QRS complexes illustrated by red arrows. <5mm (5 small squares) in limb leads and <10mm (10 small squares) in precordial leads. (B) Lead II rhythm strip demonstrating electrical alternans (alternating QRS complex sizes) due to movement of the heart with the large pericardial effusion. Large complexes (red arrows). Smaller complexes (green arrows) This image was created by the authors using PMcardio.com (Powerful Medical, New York, NY, USA).

The 12-lead ECG is obtained almost universally in patients with chest pain, but its role is limited in diagnosing pericardial effusions, especially without pericarditis. Pericarditis changes include PR segment depression, global saddle-shaped ST elevation, and the absence of Q waves. Changes, such as low-voltage QRS complexes or electrical alternans, are commonly seen only in large pericardial effusions (Figure 3A). Electrical alternans refer to alternating voltages of high and low QRS complexes that are explained by swinging motion, and therefore an alternating cardiac axis, of the heart within the fluid (Figure 3B). These findings are specific to the presence of pericardial effusion but come with low sensitivity [18].

Chest Radiography

**Figure 4:**
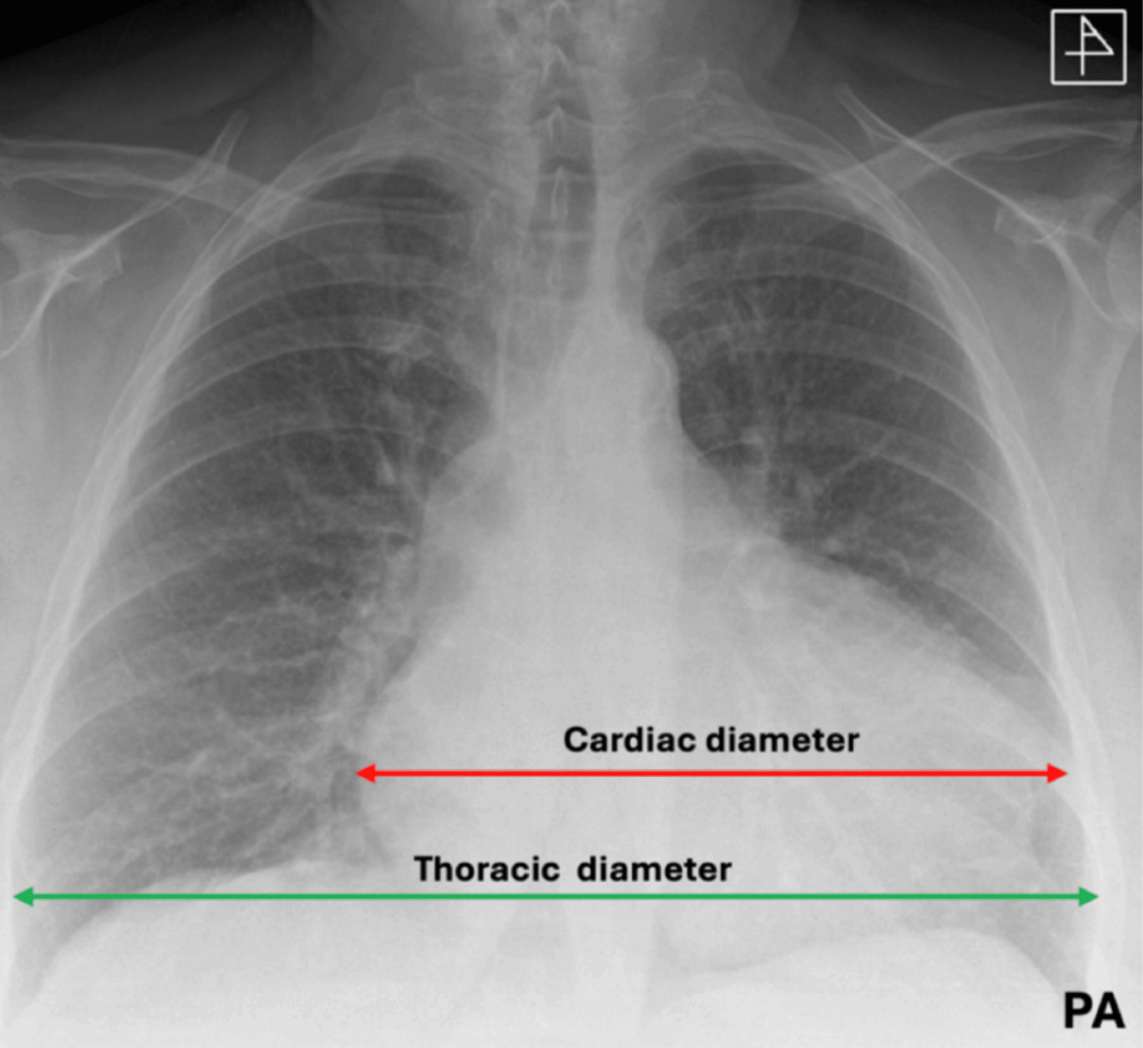
PA chest X-ray film demonstrating enlargement of the cardiac shadow. Cardiac diameter (red arrow) is more than 50% of the thoracic diameter (green arrow) This image has not been published elsewhere. PA: posterior-anterior

The plain chest X-ray is commonly performed in cases of dyspnoea and chest pain. Still, it is of limited use in the diagnosis of pericardial effusion, as noted by Eisenberg et al. [19], who found that an enlarged cardiac silhouette was only 71% specific and 41% sensitive for pericardial effusion. Clinically significant changes are only visible when there is an effusion of 300 ml, causing a global, often flask-shaped, enlargement of the cardiac silhouette (Figure 4) [20]. The lung fields themselves, however, will be typically clear.

Echocardiography

**Figure 5:**
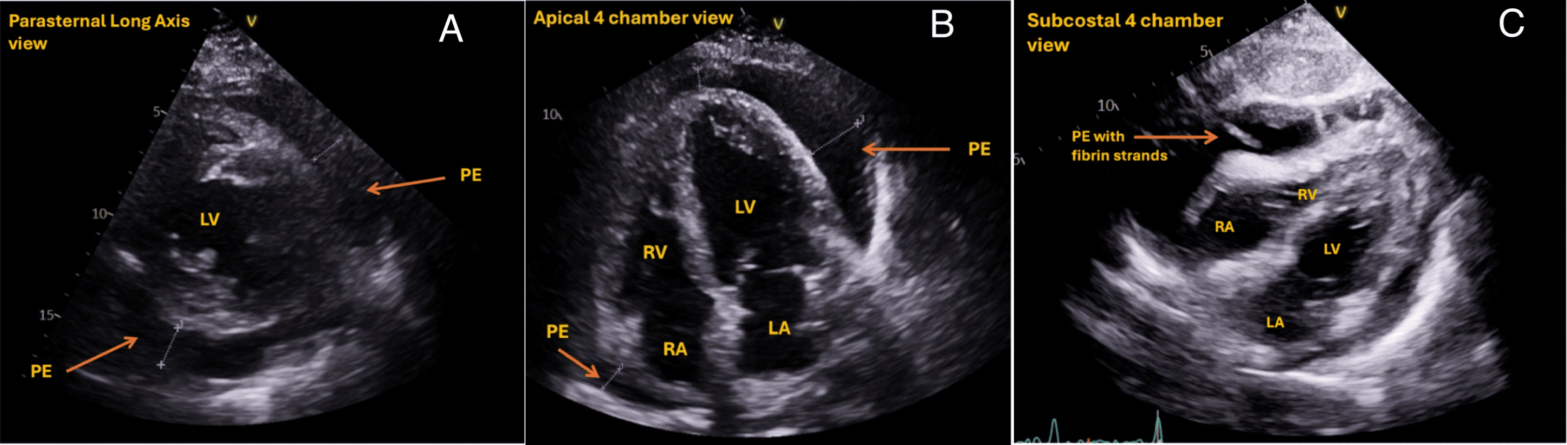
Presence of pericardial effusion on TTE. Effusion marked by the orange arrow. (A) Parasternal long axis view. (B) Apical four-chamber view. (C) Subcostal four-chamber view of TTE illustrating the presence of fibrin strands within the pericardial effusion TTE: transthoracic echocardiogram, PE: pericardial effusions, LV: left ventricle, RV: right ventricle, LA: left atrium, RA: right atrium This image has not been published elsewhere.

TTE remains the mainstay (Class IC recommendation) of diagnosis with respect to pericardial effusions [21]. On TTE, pericardial effusion appears as an anechoic between the layers of pericardium (Figure 5). Ideally, the pericardial effusion should be demonstrated in multiple views, paying particular attention to the depth (measured during diastole) and location, e.g. distribution (Table 5). Whilst cardiac tamponade is a clinical diagnosis, there are certain TTE-based signs of cardiac compromise and haemodynamic instability (Table 6). It is crucial to emphasise that the absolute volume of effusion has no haemodynamic significance or correlation with symptoms, but factors influencing this can be found in Table 7. Lastly, TTE plays a role in providing a qualitative assessment of the effusion to determine its aetiology, such as identifying fibrin strands, which may suggest malignancy or infection as a cause (Figure 5C). Recent advancements include the integration of real-time 3D echocardiography, which enhances spatial visualisation of effusions and anatomy to improve diagnostic accuracy and procedural guidance.

**Table 5:** Classification of pericardial effusions This table was created by the authors.

Category	Description	Definition
Onset	Acute	<3 months
	Chronic	>3 months
Depth/size	Mild moderate large	<10 mm, 10-20 mm, >20 mm
Distribution	Localized global	Adjacent to ≥1 cardiac chamber, adjacent to all four cardiac chambers
Composition	Exudate transudate	Protein >30g/L, protein <30g/L

**Table 6:** Echocardiographic signs of cardiac tamponade This table was created by the authors.

Feature	Explanation
Large pericardial effusion with swinging heart	Echocardiographic equivalent of electrical alternans
Diastolic collapse of the right atrium or ventricle	Pericardial pressure > right atrial or ventricular pressure
Inferior vena cava plethora	Pericardial pressure > right atrial and therefore reduced venous return
Respiratory changes of mitral or tricuspid valve inflow	Doppler surrogate of pulsus paradoxus

**Table 7:** Factors influencing the magnitude of haemodynamic abnormalities This table was created by the authors.

	Increase	Decrease
Rate of pericardial fluid accumulation	Rapid, e.g. minutes-hours	Slow, e.g. weeks-months
Pericardial compliance	Stiff, e.g. constrictive pericarditis	Pliable, e.g. normal pericardium
Intracardiac filling pressures	Low, e.g. normal health	High, e.g. left ventricular hypertrophy/left ventricular systolic dysfunction/hypertension/aortic stenosis/heart failure with preserved ejection fraction or Right ventricular hypertrophy/right ventricular systolic dysfunction/pulmonic stenosis/pulmonary arterial hypertension
Intracardiac compliance	Low, e.g. normal myocardium	High, e.g. left ventricular hypertrophy or right ventricular hypertrophy

Furthermore, artificial intelligence (AI)-based image analysis is increasingly applied to TTE and other imaging modalities. This facilitates assessment and improves detection of subtle abnormalities [22]. However, the discussion of the use of AI and imaging within pericardial disease is beyond the scope of this review.

Computed Tomography

**Figure 6:**
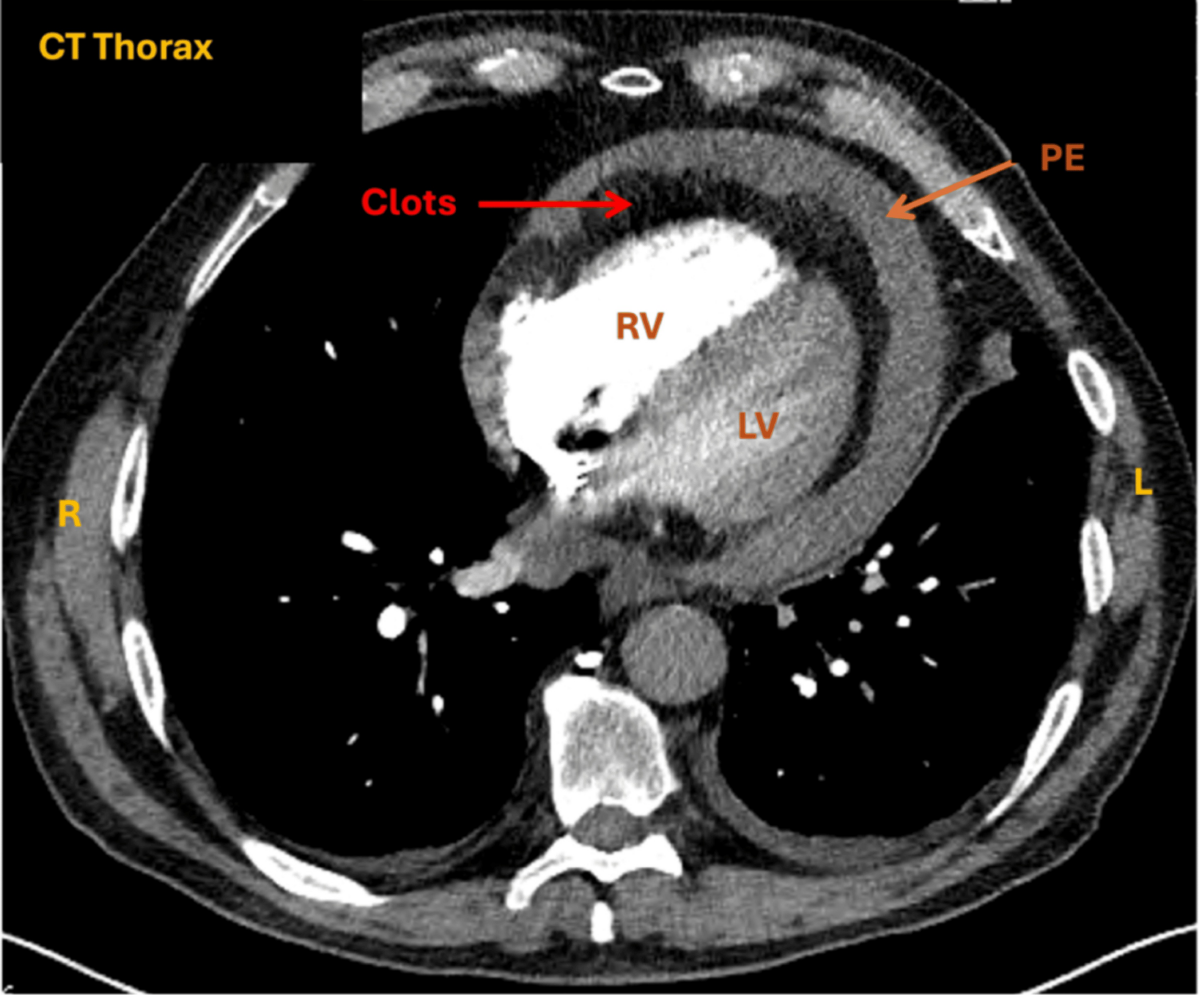
Contrast CT thorax demonstrating presence of pericardial effusion (orange arrow) CT: computed tomography, PE: pericardial effusions, LV: left ventricle, RV: right ventricle This image has not been published elsewhere.

Computed tomography (CT) scan serves as a complementary modality to TTE (Class IC recommendation [21]). It is particularly valuable in the localisation of complex or loculated effusions and differentiating fluid from clot (Figure 6). In clinical practice, it is more common for an incidental pericardial effusion to be noted on a CT pulmonary angiogram or CT chest, prompting confirmatory TTE. CT also aids in identifying extracardiac pathology, such as cancer, and information on the composition (transudative vs. exudative) of the effusion based on CT attenuation values. However, cardiac MRI and TTE are superior to CT in identifying effusions due to pericardial thickening.

Cardiac Magnetic Resonance Imaging

**Figure 7:**
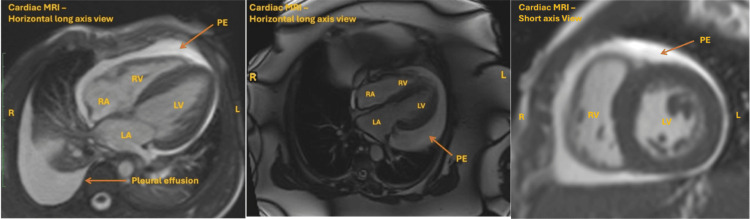
Different views of cardiac MRI demonstrating pericardial effusion. Effusion marked by the orange arrow. (A & B) Horizontal long axis view. (C) Short axis view MRI: magnetic resonance imaging, PE: pericardial effusions, LV: left ventricle, RV: right ventricle, LA: left atrium, RA: right atrium This image has not been published elsewhere.

Cardiac MRI (Class IC recommendation) excels in detecting small and loculated effusions that are not evident on a TTE and provides a detailed assessment of the degree of inflammation of the pericardium (Figure 7). It may be used to evaluate the response to treatment, especially when patients present with recurrent pericarditis, and help decide the further course of management, i.e. tapering of steroids [23]. However, cardiac MRI should ideally be reserved for the chronic effusions in stable patients where the aetiology is unclear.

Pericardial Fluid Analysis

There is no formal class designation for pericardial fluid analysis within the European Society of Cardiology (ESC) guidelines. Still, it is considered standard practice when the diagnosis is unclear; pericardial fluid samples are typically sent for chemistry, cytology, microscopy, and culture, as well as biomarkers, polymerase chain reaction, histology, acid-fast bacilli, and glucose in specific cases. Nearly two-thirds of malignancy-related effusions may not contain malignant cells, so further imaging, such as CT or CT positron emission tomography, will be necessary if malignancy is suspected [3].

Treatment

The treatment of pericardial effusions largely depends on identifying the underlying cause. Besides the aetiology, other essential factors that influence management include the chronicity of the effusion, its haemodynamic effects, and its distribution, i.e. circumferential, loculated, or global. Treatment can be divided into medical and invasive management.

Medical Management

The mainstay treatment of pericardial effusion is to treat the underlying condition. This could include treating infectious causes with antimicrobial agents, heart failure with diuretics, and dialysis for renal failure. Treating pericardial effusions secondary to pericarditis with non-steroidal anti-inflammatory agents (NSAIDs) is beneficial [21]. 2025 ESC guidelines recommend starting NSAIDs only if the CRP is elevated [24].

In a prospective study done in patients with chronic, idiopathic, large PE in asymptomatic patients, albeit a small study of 61 patients, Lazarou et al. noted that the complication-free survival rate was not significantly different between patients undergoing pericardiocentesis and those treated conservatively [25].

Recently, IL-6 inhibitors such as Anakinra and IL-1 Rilonacept have been used for treating recurrent pericarditis with or without effusion with good effect, especially in those who have resistant disease [26,27]. Both these medications act rapidly and have a good side effect profile and are ideally used as third-line following NSAIDs, colchicine and steroids [26].

Malignant effusions may be treated with intrapericardial sclerosing agents such as colchicine, bleomycin, doxycycline, tetracycline, and cisplatin [1]. This will induce inflammatory changes in the pericardial layers, which will eventually lead to adhesions and reduce reaccumulation [1]. In a literature review, Jama et al. noted a success rate of 87.8% but suffered from a high rate of morbidity (20.5%) [28].

Invasive Management

Pericardiocentesis: According to the ESC algorithm for managing pericardial effusion, pericardiocentesis is advised for patients with cardiac tamponade or suspected bacterial or neoplastic causes [21]. Furthermore, if there is a large effusion accompanied by symptoms but without evidence of tamponade, a pericardiocentesis should be considered for symptomatic relief as well as to aid with diagnosis. However, pericardiocentesis is not without risk (morbidity 3% and mortality <1%), and draining of pericardial effusion without any high-risk or tamponade features has a low diagnostic yield [18]. Ristic et al. suggested using a scoring system to decide the time frame within which pericardiocentesis needs to be performed [29].

Typically, for uncomplicated effusions that require drainage, pericardiocentesis is performed under echocardiographic and/or fluoroscopic guidance. The subcostal approach is considered the safest route; however, the approach should be based on the distribution of the effusion, and apical or parasternal approaches are also used. Insertion of the pericardial drain is done using the Seldinger technique. In brief, the needle with a syringe attached is inserted under continuous visualisation by TTE, and fluid is aspirated as the needle is advanced. Once the needle enters the pericardium, the instillation of saline helps confirm the location and serves as supplementary evidence. This is followed by the insertion of a guidewire, removal of the needle, dilation of the puncture tract, and finally, the insertion of the catheter, which is secured using sutures. The pericardial fluid is aspirated until signs of tamponade have subsided. Typically, a drain is removed after 24 hours or if it is no longer actively draining.

Pericardiocentesis carries a risk between 4% and 10%, depending upon the experience of the operator, monitoring available, and the setting, i.e. urgent vs. elective [24]. Complications are grouped into major and minor categories. Major complications include damage to surrounding organs such as the liver and lungs, perforation of the peritoneum or abdominal viscera, pneumothorax, pericardial decompensation, pneumopericardium, cardiac or coronary perforation, and arrhythmias. Minor complications include infection, minor bleeding, pain, and vasovagal syncope.

Pericardial window: Surgical evacuation may be attempted via a pericardial window or pericardiotomy if the effusion is secondary to trauma or post-cardiac surgery, or if the loculated effusion is causing effects of tamponade or is posterior, e.g. difficult to reach with a pericardiocentesis needle. Pericardial window surgery involves surgically creating a conduit between the pericardial space and the pleural space to facilitate fluid drainage. The procedure is typically performed by a cardiothoracic surgeon using video-assisted thoracoscopic surgery or via balloon pericardiotomy through a percutaneous approach. This surgery is usually done as a palliative measure to provide symptomatic relief. In a retrospective analysis conducted by Lee et al. on patients with malignant pericardial effusion, it was noted that pericardial window surgery significantly reduced the need for repeated invasive interventions [30].

Special Population

Pregnancy: Forty per cent of pregnant patients may have mild pericardial effusion. In a small study done by Dennis and Castro, 73% of the patients with severe pre-eclampsia had pericardial effusion [31]. Typically, small effusions do not require treatment and usually resolve spontaneously after delivery [31,32]. Pregnant patients with pericarditis may be treated like non-pregnant patients except that NSAIDs are not used beyond the 20th week and require involvement of the multidisciplinary team [32].

HIV: Patients with lower CD counts are at higher risk of developing pericardial effusion [33]. It is estimated that 9% of HIV patients can develop pericardial effusion, and nearly 1% of these can develop tamponade [33]. Infection and malignancy are the leading causes of pericardial effusion in HIV patients. Pathogens like *Staphylococcus aureus*, *Streptococcus* species, *Klebsiella*, *Mycobacterium*, *Cryptococcus neoformans*, *Nocardia asteroids*, *Aspergillus species*, *Cytomegalovirus,* and herpes simplex virus have been identified [33]. Malignancies such as lymphoma, Kaposi’s sarcoma, and adenocarcinoma are the leading causes. Patients with features and signs of cardiac tamponade need pericardiocentesis. Kwan et al. noted that 54% of the patients died at the time of discharge, whilst Keyvanfar et al. noted in their systematic review that 80.55% of the patients recovered after appropriate treatment [33,34].

Malignancy: Patients with pericardial effusions and normal inflammatory markers have an increased risk of neoplasm as the aetiology (likelihood ratio of 2.9) [24]. The patient may be asymptomatic. Metastatic disease is more commonly associated with pericardial effusions than primary cardiac disease. Solid tumours such as lung and breast cancers are commonly identified as causes, followed by lymphoma, leukaemia, and gastrointestinal malignancies [35]. Systemic anti-cancer treatment, radiotherapy, sclerotherapy, and fluid drainage either by pericardiocentesis or surgical window remain the mainstay of treatment. Median age of survival is between 7 and 15 weeks for patients with pericardial effusion secondary to malignancy [36]. More patients are being treated with immunotherapy checkpoint inhibitors. Pericardial effusions secondary to an immune checkpoint inhibitor are a rare but serious adverse reaction [37]. It can typically be treated with NSAIDs, colchicine, and corticosteroids [35,37].

Radiotherapy can also lead to pericardial effusion, presenting either acutely or with a delayed onset. It occurs because the heart and left side of the thorax are in the field of irradiation. The contributing factors are generally categorised as patient-related or radiotherapy-related [35]. Patient-related factors include older age, left-sided tumours, and the presence of diabetes [35]. Radiotherapy-related factors include receiving a dose exceeding 50 Gy and irradiation of a larger portion of the pericardium [35].

Prognosis

The prognosis of pericardial effusions is widely based on the underlying cause, speed, and size of accumulation. Idiopathic and viral effusions and pericarditis generally have a benign prognosis. Whereas bacterial or fungal infections carry a high mortality rate and poor prognosis. A malignant pericardial effusion may suggest that the underlying cancer is in the advanced stages, which carries a poor prognosis. The prognosis of effusions caused by rheumatological aetiologies depends on the underlying disease. Overall, the prognosis is favourable when an effusion is identified and there is prompt definitive management of the underlying cause. In a meta-analysis conducted by de Filippo et al., pericardial effusion was identified as a marker of disease severity. In contrast, in idiopathic cases, its correlation with poor prognosis was less clear [38]. In a study involving 100 patients, Imazio et al. reported a risk of tamponade of 2.2% per year, with better survival outcomes for patients who were managed conservatively [39].

Follow-up

For mild effusions <10 mm, typically, there is no specific follow-up interval required, provided that the cause has been identified and treated. [21] Moderate effusions between 10 and 20 mm in depth require a repeat TTE every six months until successful treatment or spontaneous resolution [21]. Large effusions >20 mm in depth will require repeat TTE every three months after successful treatment or spontaneous resolution, as such large chronic effusions (>3 months) have a 30-35% risk of progression to cardiac tamponade [21].

However, according to the new ESC guidelines from 2025, in the absence of symptoms, patients with mild idiopathic effusions do not require any follow-up [24]. Patients with at least moderate effusions without symptoms should be followed up every six months, ideally in a specialised centre [24]. Patients need to be safety-netted and advised to seek urgent help [24].

## Conclusions

The most important role of a clinician is to identify the likely aetiology of the effusion, assess for clinical evidence of haemodynamic instability and then consider the appropriate management. The causes of a pericardial effusion are legion, and therefore, careful history taking is required to delineate the probable causes. Lastly, long-term surveillance with serial TTE assessment coupled with clinical surveillance enables early detection of progression and guides prompt management.

Key points highlight the importance of a thorough history taking and diagnostic work-up to determine the underlying aetiology. TTE is the most useful initial qualitative and quantitative imaging tool to assess pericardial effusion. The rate of fluid accumulation is more concerning than the absolute volume present. A hallmark feature of cardiac tamponade is pulsus paradoxus. Ultimately, treatment depends on both the underlying aetiology and the haemodynamic status of the patient.
